# Fostering international collaboration in implementation science and research: a concept mapping exploratory study

**DOI:** 10.1186/s13104-019-4800-4

**Published:** 2019-11-29

**Authors:** Gregory A. Aarons, Chariz Seijo, Amy E. Green, Joanna C. Moullin, Henna Hasson, Ulrica von Thiele Schwarz, Sigrid James, Mark G. Ehrhart, Simon Ducarroz, Nick Sevdalis, Cathleen Willging

**Affiliations:** 1 Department of Psychiatry, University of California San Diego, 9500 Gilman Drive (0812), La Jolla, CA 92093-0812 USA; 2 Child and Adolescent Services Research Center, 3665 Kearny Villa Rd., Suite 200N, San Diego, CA 92123 USA; 3 School of Pharmacy, Department of Health Sciences, Curtin University, Kent Street, Bentley, Perth, WA 6102 Australia; 4 Medical Management Centre, Dept. of Learning, Informatics, Management and Ethics, Karolinska Institutet, 171 77 Stockholm, Sweden; 5 ​School of Health, Care and Social Welfare, Mälardalen University, 721 23 Västerås, Sweden; 6 Department of Social Work and Social Welfare, University of Kassel, Arnold-Bode Str. 10, 34127 Kassel, Germany; 7 Department of Psychology, University of Central Florida, 4111 Pictor Lane, Orlando, FL 32816-1390 USA; 8 EA 7425 HESPER-Health Services and Performance Research, Université Claude Bernard Lyon 1, 8 Av. Rockefeller, Domaine Rockefeller- 2ème Étage (aile CD), 69373 Lyon Cedex 8, France; 9 Center for Implementation Science, Institute of Psychiatry, Psychology & Neuroscience (IoPPN), King’s College London, 16 De Crespigny Park, London, SE5 8AF UK; 10 Behavioral Health Research Center of the Southwest, Albuquerque, NM USA

**Keywords:** Implementation, Dissemination, International, Collaboration, Team science, Concept mapping, Qualitative, Quantitative, Mixed-methods

## Abstract

**Objective:**

International collaboration in science has received increasing attention given emphases on relevance, generalizability, and impact of research. Implementation science (IS) is a growing discipline that aims to translate clinical research findings into health services. Research is needed to identify efficient and effective ways to foster international collaboration in IS. Concept-mapping (CM) was utilized with a targeted sample for preliminary exploration of fostering international collaboration. Concept-mapping is a mixed-method approach (qualitative/quantitative) particularly suited for identifying essential themes and action items to facilitate planning among diverse stakeholders. We sought to identify key factors likely to facilitate productive and rewarding international collaborations in implementation research.

**Results:**

We identified eleven dimensions: Strategic Planning; Practicality; Define Common Principles; Technological Tools for Collaboration; Funding; Disseminate Importance of Fostering International Collaboration in IS; Knowledge Sharing; Innovative & Adaptive Research; Training IS Researchers; Networking & Shared Identity; Facilitate Meetings. Strategic Planning and Funding were highest rated for importance and Strategic Planning and Networking and Shared Identity were rated most feasible to institute. Fostering international collaboration in IS can accelerate the efficiency, relevance, and generalizability of implementation research. Strategies should be developed and tested to improve international collaborations and engage junior and experienced investigators in collaborations advancing implementation science and practice.

## Introduction

Implementation science (IS) aims to translate research findings into health and social care. IS has been defined as “the scientific study of the use of strategies to adopt and integrate evidence-based health interventions into clinical and community settings in order to improve patient outcomes and benefit population health [1].” IS addresses the healthcare research-to-practice gap by assessing and improving the adoption of evidence-based practices (EBPs; i.e., those with proven effectiveness) into real-world settings [2]. The research-to-practice gap remains a concern: for instance, as little as 2% of individuals with serious mental illness using publicly funded services in the US received evidence-based care [3]. Researchers in multiple countries and across several disciplines increasingly recognize and use IS approaches and processes to improve healthcare by testing strategies to facilitate EBP implementation and sustainment in high income as well as low and middle income countries (LMICs) [4–9].

As a relatively new discipline, key theoretical frameworks strategies, theories, and measures are still being developed and refined [4, 10, 11]. To address the various challenges of translating research findings into practice, researchers must develop new approaches by incorporating knowledge from diverse perspectives [12]. Collaborations between implementation researchers working in different contexts can contribute to the development of novel and generalizable approaches. In an increasingly globalized world, collaborations must go beyond traditional boundaries to address barriers such as geopolitical and cultural differences and capitalize on shared learning and perspectives. Effective approaches and interventions may be discovered and advanced through cross-disciplinary collaborations in science [13–15].

Collaborations of implementation researchers may foster a greater understanding of the common and unique challenges in IS in various settings, and the strategies necessary to successfully improve quality of care and implementation and sustainment of evidence-based practices within multi-layered social contexts [6, 16]. Because IS draws from many areas of research and aims to solve a diverse range of problems, collaboration is essential to advance the state of the field. However, little is known about the conditions that may facilitate international collaborations in IS.

## Main text

The purpose of this study was to garner multiple perspectives on fostering international collaboration in implementation science (FICIS). This study employed concept mapping (CM), a mixed-methods (qualitative and quantitative) approach for data collection and analysis that incorporates input from all participants in order to identify dimensions of productive collaboration and assess their importance and feasibility for FICIS.

### Participants

Ten implementation researchers participated in a 3-day retreat to foster productive international collaborations in IS. Participants were selected to represent different implementation contexts (e.g., US, Europe) and health issues (e.g., mental health, cancer, occupational health, social care). The participant countries were as follows: France, Germany, the United Kingdom, Australia, Sweden, and the US. All participants had experience in implementation research in those countries as well as collaborations in Spain, Norway, Switzerland, Belgium, Colombia, Mexico, Nigeria, and Sierra Leone. Academic disciplines represented included epidemiology, anthropology, occupational health, social work, pharmacy, business management, organizational psychology, and clinical psychology. Participants represented several domains in health and human services including behavioral health, school-based care, social services, occupational health, nursing, pharmacy, and medicine. The mean number of years of experience in IS was 8.1 years (range = 1.5–18 years), and the mean number of years in international experience was 2.9 years (range = 0.3–5.5 years). Of the 10 participants, eight were professors/faculty and two were post-doctoral scientists.

Concept Mapping consists of six phases: (1) preparation, identify stakeholder participants and collaboratively develop a focus question; (2) generation, participants brainstorm responses to the focus question; (3) structuring, participants sort statements based on similarity and rate statements on a priori dimensions (e.g., importance, feasibility); (4) representation, researchers conduct multidimensional scaling (MDS) and cluster analyses to create a “concept map”; (5) interpretation, researchers/participants collaboratively develop cluster labels and interpretations; and (6) utilization, researchers/participants use results to identify action-items and next-steps. In CM small samples may be adequate and data from at least 10 participants can produce reliable results [17, 18].

Three primary outputs were used: (1) Cluster map identifying the most important dimensions or clusters representing each concept, (2) Cluster ranking based on ratings of importance and feasibility, (3) Pattern matching that shows and correlates the relative ranking for cluster importance and feasibility.

Participants jointly and iteratively developed a single focal question: “What are the ways to foster international collaboration in implementation science in health and social care?” In the generation phase, brainstorming occurred in-person collectively through a group process. Multiple responses from each participant were elicited. Ten participants contributed 61 unique statements regarding FICIS. In the structuring phase, participants used online software and individually sorted the statements into separate groups (or “clusters”) in a manner that was meaningful to them. Finally, participants individually rated each statement on importance: “How important is this factor for fostering international collaboration in implementation science (FICIS)?” (0 = not at all; 5 = to a very great extent), and feasibility: “How feasible is this factor for FICIS?” (0 = not at all; 5 = to a very great extent).

### Analyses

Statement sorting data were analyzed using MDS and hierarchical cluster analysis. These procedures resulted in the visual representations (i.e., concept maps) for how statements were typically clustered across all participants. Multiple CM outcomes were considered based on acceptable overall “stress” fit statistic and interpretability of each potential solution. The “stress value” of the point map is a measure of how well the MDS solution maps the original data. The stress value is derived from normalized residual variance for a perfect relationship of a regression of the distance of dissimilarity or similarity. The range for CM has been reported as 0.21–0.37, with lower stress values reflecting better fit of the MDS point map to the original data [19]. The ideal model would include the fewest number of clusters that also retained distinct themes. This process considered a larger number of potential thematic clusters (e.g., 14) and then, in a stepwise fashion, consolidating clusters that were thematically similar based on participant responses. These models were reviewed by the study team with the final concept map (11 thematic clusters) approved by consensus of all authors. Each cluster in the final model was collaboratively named to reflect the content contained in each cluster. All participants reviewed the model and gave feedback on the final results. We also examined “Go Zone” maps that place each statement in a two-dimensional space with the Y axis indicating importance rating and the X axis representing feasibility.

## Results

**Fig. 1:**
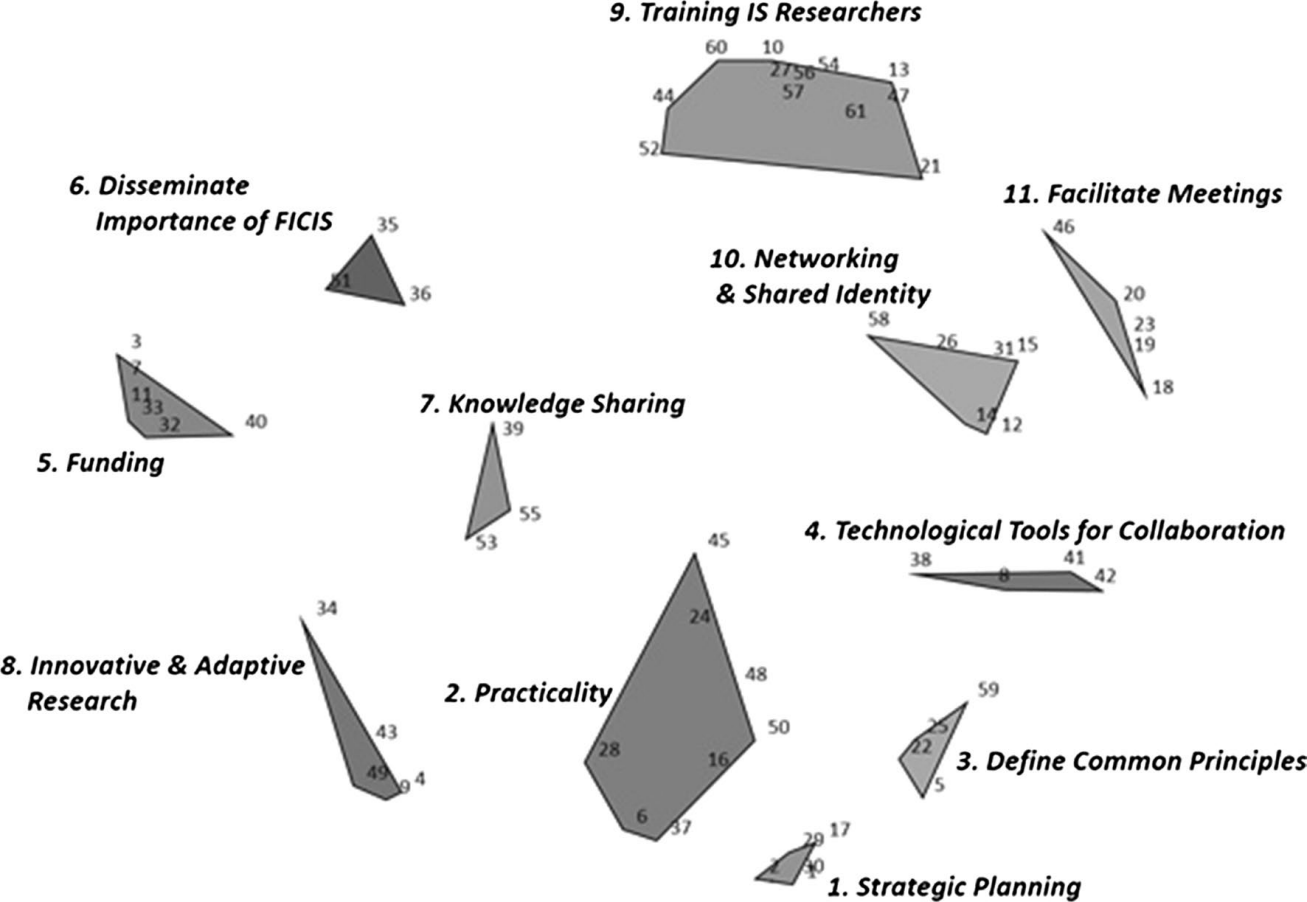
Eleven Cluster Thematic Concept Mapping Solution (stress value = 0.30)

The final model comprised 11 thematic clusters, each representing a key domain of FICIS, with a stress value of 0.30 indicating adequate fit (Fig. 1). These clusters were: *Strategic Planning; Practicality; Define Common Principles; Technological Tools for Collaboration; Funding; Disseminate Importance of FICIS; Knowledge Sharing; Innovative & Adaptive Research; Training IS Researchers; Networking & Shared Identity; Facilitate Meetings.* Each number within a cluster represents a statement that was sorted into similar categories by participants.

**Fig. 2:**
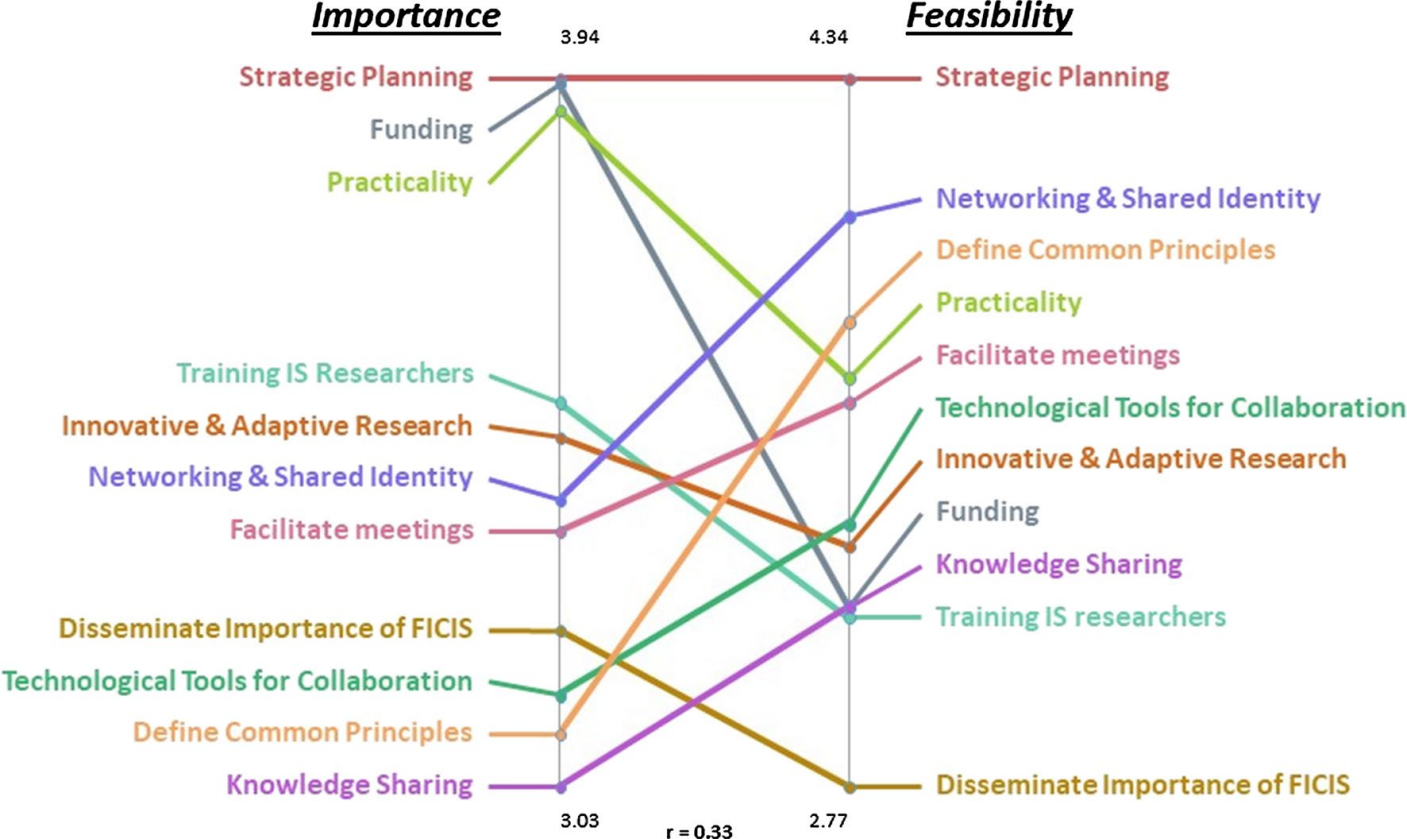
Pattern Match 11 Cluster Solution with Importance and Feasibility Ratings

Table 1 shows the mean participant ratings for each cluster and the rank order for the ratings of (1) importance for FICIS and (2) feasibility for FICIS. The “importance” ratings for FICIS ranged from a low of 3.03 (Knowledge Sharing) to a high of 3.94 (Strategic Planning) on the 5-point scale. This demonstrates relatively higher importance for developing a strategic plan compared to sharing knowledge among international collaborators. Similarly, the “feasibility” cluster ratings for FICIS ranged from a low of 2.77 (Disseminate Importance of FICIS) to a high of 4.24 (Strategic Planning). This suggests that Strategic Planning may be more feasible than Disseminating the Importance of FICIS: Table 1 also provides a detailed cluster-by-cluster comparison for the ratings and relative ranking across these two dimensions. The far right column shows the combined scores and ranking of the two dimensions. This was done in order to show and suggest what factors might be both important and feasible. Figure 2 shows the “pattern match” results that provides another perspective and shows both the importance ratings on the left and feasibility ratings on the right. The correlation between Importance and Feasibility ratings was *r* = 0.33, indicating a medium effect size. There is some variability in the clusters determined to be among the most important and the most feasible for FICIS. For example, results indicate that the thematic cluster, Strategic Planning (e.g., structured plan for collaboration, goals, products), was ranked the top cluster for both dimensions indicating that it is a factor that is both important, but also can be instituted relatively easily. In contrast, Funding was rated highly important but low on feasibility. The clusters for Funding (e.g., be proactive about pursuing funding opportunities for joint knowledge exchange/production) and Practicality (e.g., identify collaborators with common interests) rounded out the top three themes determined most important for FICIS. Networking & Shared Identity (e.g., joint symposia presentations with presenters from multiple countries) and Define Common Principles (e.g., promote principles of diversity and inclusion in international IS) completed the top three for feasibility.

**Table 1:** Cluster rating averages and ranks for Fostering International Collaboration in Implementation Science (FICIS)

Thematic cluster	Importance for FICIS			Feasibility for FICIS		Combined rank
	Cluster number	Cluster rating	Cluster rank	Cluster rating	Cluster rank	Rank score
Strategic planning	1	3.94	1	4.24	1	2
Practicality	2	3.90	3	3.68	4	7
Networking and shared identity	10	3.40	6	4.03	2	8
Funding	5	3.93	2	3.17	8	10
Innovative and adaptive research	8	3.48	5	3.30	7	12
Facilitate meetings	11	3.36	7	3.62	5	12
Define common principles	3	3.10	10	3.80	3	13
Training is researchers	9	3.53	4	3.14	10	14
Technological tools for collaboration	4	3.15	9	3.35	6	15
Disseminate importance of FICIS	6	3.23	8	2.77	11	19
Knowledge sharing	7	3.03	11	3.17	9	20

*FICIS* Fostering International Collaboration in Implementation Science. Cluster ratings ranged from 0 to 5, with higher scores indicating more importance or greater feasibility for FICIS

In the GO Zone Map (Additional file 1), all 61 individual statements are plotted by their average importance and feasibility ratings. The correlation of items across quadrants was *r* = 0.24 indicating a small to moderate correlation. Each plot point has a number that corresponds with a statement in the table (Additional file 2), which also provides the complete list of statements within each cluster. The GO Zone map is split into four quadrants with the upper right quadrant indicating statements with both above average importance and feasibility ratings and thus are considered actionable and are likely targets to be prioritized in FICIS.

## Discussion

We identified 11 domains impacting FICIS with some overlap among the most highly rated thematic clusters across the two dimensions of importance and feasibility. Strategic Planning was rated the top cluster for both importance and feasibility. This emphasizes how careful planning, such as developing goals and principles for collaboration, is an essential and achievable first step in developing long-term collaborations in IS. The next highest-rated clusters for “importance,” Funding and Practicality, were both rated as less feasible (eighth and fourth, respectively). Funding was rated the second-most important cluster but only eighth in feasibility. This is consistent with the notion that international collaboration efforts should focus on finding creative solutions supporting, coordinating, and integrating new initiatives [20].

It is also important to expand the work conducted for this study more broadly with more perspectives representing additional countries, geographic regions, and health conditions. Another approach could be engaging groups with a focus on specific levels of strategies (e.g., influencing policy, organizational change, physician behavior change, community pharmacy, etc.). In regard to funding, researchers could respond to calls for funding for health care improvement in specific settings such as LMIC, or types of health care systems (e.g., single payer, insurance markets, etc.). However, funding for research on improving the quality and delivery of services is relatively low compared to basic science research [21]. Thus, nurturing international collaborations is a key priority to accelerate IS progress.

Several specific strategies could be developed and utilized for FICIS. For example, international strategic planning groups could be established that focus on deploying IS to address specific types of health problems (e.g., cancer, obesity, etc.) that cut across borders and populations. Another approach is to organize strategic planning groups that concentrate on creating and evaluating specific implementation strategies targeting change at multiple levels (e.g., policy, organizational, community pharmacy, physician, lay health worker, etc.). International researchers must identify opportunities for funding that allows collaborations across geographic regions [22]. Such efforts should also include researchers, policy makers (e.g., health ministry) and other community stakeholders (e.g., lay health workers, patients, etc.) from LMICs (where relevant), and/or types of health care systems (e.g., socialized medicine, single payer, insurance markets, fee for service, etc.).

## Conclusions

Implementation researchers traditionally share their research experiences across diverse disciplines through meetings and conferences that lead to some collaborations with a shared goal of advancing the integration of research into practice [21]. Future international collaborations of implementation researchers can be advanced based on our results and have the potential to improve the quality and external validity of implementation research, develop new and early career researchers, and expand the scope and network of engaged implementation scientists.

## Limitations

For this particular project the participants were primarily conducting research in developed countries, with less representation of implementation research in LMICs. Work is under way to expand the purview and international representation of this line of work. Future work should include a larger number of respondents of more nationalities and conducting implementation research in other geographical and system settings including LMICs.

## Supplementary information

**Additional file 1.** GO Zone map.

**Additional file 2.** List of statements within each cluster.

## Data Availability

The datasets used and/or analyzed during the current study are available from the corresponding author on reasonable request.

## Abbreviations

CM: concept mapping
FICIS: fostering international collaboration in implementation science
IS: implementation science
LMICs: low and middle income countries
MDS: multidimensional scaling
US: United States

## References

[1] NIH. Dissemination and implementation research in health (R01 Program Announcement) 2016. http://grants.nih.gov/grants/guide/pa-files/PAR-16-238.html.

[2] Brownson RC, Jones E (2009). Bridging the gap: translating research into policy and practice. Prev Med.

[3] Bruns EJ, Kerns SE, Pullmann MD, Hensley SW, Lutterman T, Hoagwood KE (2016). Research, data, and evidence-based treatment use in state behavioral health systems, 2001–2012. Psychiatr Serv.

[4] Aarons GA, Hurlburt M, Horwitz SM (2011). Advancing a conceptual model of evidence-based practice implementation in public service sectors. Adm Policy Ment Health..

[5] Aarons GA, Green AE, Palinkas LA, Self-Brown S, Whitaker DJ, Lutzker JR (2012). Dynamic adaptation process to implement an evidence-based child maltreatment intervention. Implement Sci..

[6] Glisson C, Schoenwald S (2005). The ARC organizational and community intervention strategy for implementing evidence-based children’s mental health treatments. Ment Health Serv Res..

[7] von Thiele Schwarz U, Hasson H (2011). Employee self-rated productivity and objective organizational production levels: effects of worksite health interventions involving reduced work hours and physical exercise. J Occup Environ Med.

[8] Peltzer K, Prado G, Horigian V, Weiss S, Cook R, Sifunda S (2016). Prevention of mother-to-child transmission (PMTCT) implementation in rural community health centres in Mpumalanga province, South Africa. J Psychol Afr.

[9] Stetler CB, Legro MW, Wallace CM, Bowman C, Guihan M, Hagedorn H (2006). The role of formative evaluation in implementation research and the QUERI experience. J Gen Intern Med.

[10] Rabin BA, Brownson RC, Haire-Joshu D, Kreuter MW, Weaver NL (2008). A glossary for dissemination and implementation research in health. J Public Health Manag Pract..

[11] Moullin JC, Dickson KS, Stadnick NA, Rabin B, Aarons GA (2019). Systematic review of the Exploration, Preparation, Implementation, Sustainment (EPIS) framework. Implement Sci..

[12] Zerhouni E (2003). Medicine. The NIH roadmap. Science..

[13] Hiatt RA, Breen N (2008). The social determinants of cancer: a challenge for transdisciplinary science. Am J Prev Med.

[14] Stokols D, Misra S, Moser RP, Hall KL, Taylor BK (2008). The ecology of team science: understanding contextual influences on transdisciplinary collaboration. Am J Prev Med.

[15] Harris JK, Provan KG, Johnson KJ, Leischow SJ (2012). Drawbacks and benefits associated with inter-organizational collaboration along the discovery-development-delivery continuum: a cancer research network case study. Implement Sci..

[16] Aarons GA, Ehrhart MG, Farahnak LR, Sklar M (2014). Aligning leadership across systems and organizations to develop a strategic climate for evidence-based practice implementation. Annu Rev Public Health.

[17] Jackson KM, Trochim WMK (2002). Concept mapping as an alternative approach for the analysis of open-ended survey responses. Organ Res Methods..

[18] Trochim WMK. The reliability of concept mapping. Annual Conference of the American Evaluation Association1993.

[19] Green AE, Fettes DL, Aarons GA (2012). A concept mapping approach to guide and understand dissemination and implementation. J Behav Health Serv Res..

[20] Hall KL, Feng AX, Moser RP, Stokols D, Taylor BK (2008). Moving the science of team science forward: collaboration and creativity. Am J Prev Med.

[21] Glasgow RE, Vinson C, Chambers D, Khoury MJ, Kaplan RM, Hunter C (2012). National Institutes of Health approaches to dissemination and implementation science: current and future directions. Am J Public Health.

[22] Aarons GA, Sommerfeld DH, Chi BH, Ezeanolue EE, Sturke R, Guay L (2016). Concept mapping of PMTCT implementation challenges and solutions across 6 sub-Saharan African countries in the NIH-PEPFAR PMTCT implementation science alliance. J Acquir Immune Defic Syndr.

