# Supplementary material for: Fostering international collaboration in implementation science and research: a concept mapping exploratory study
Source: BMC Res Notes. 2019 Nov 29;12:778. doi: 10.1186/s13104-019-4800-4 (PMC6883596; doi:10.1186/s13104-019-4800-4)
Supplement: Supplementary file 1 — Additional file 1. GO Zone map. [file 13104_2019_4800_MOESM1_ESM.pdf]

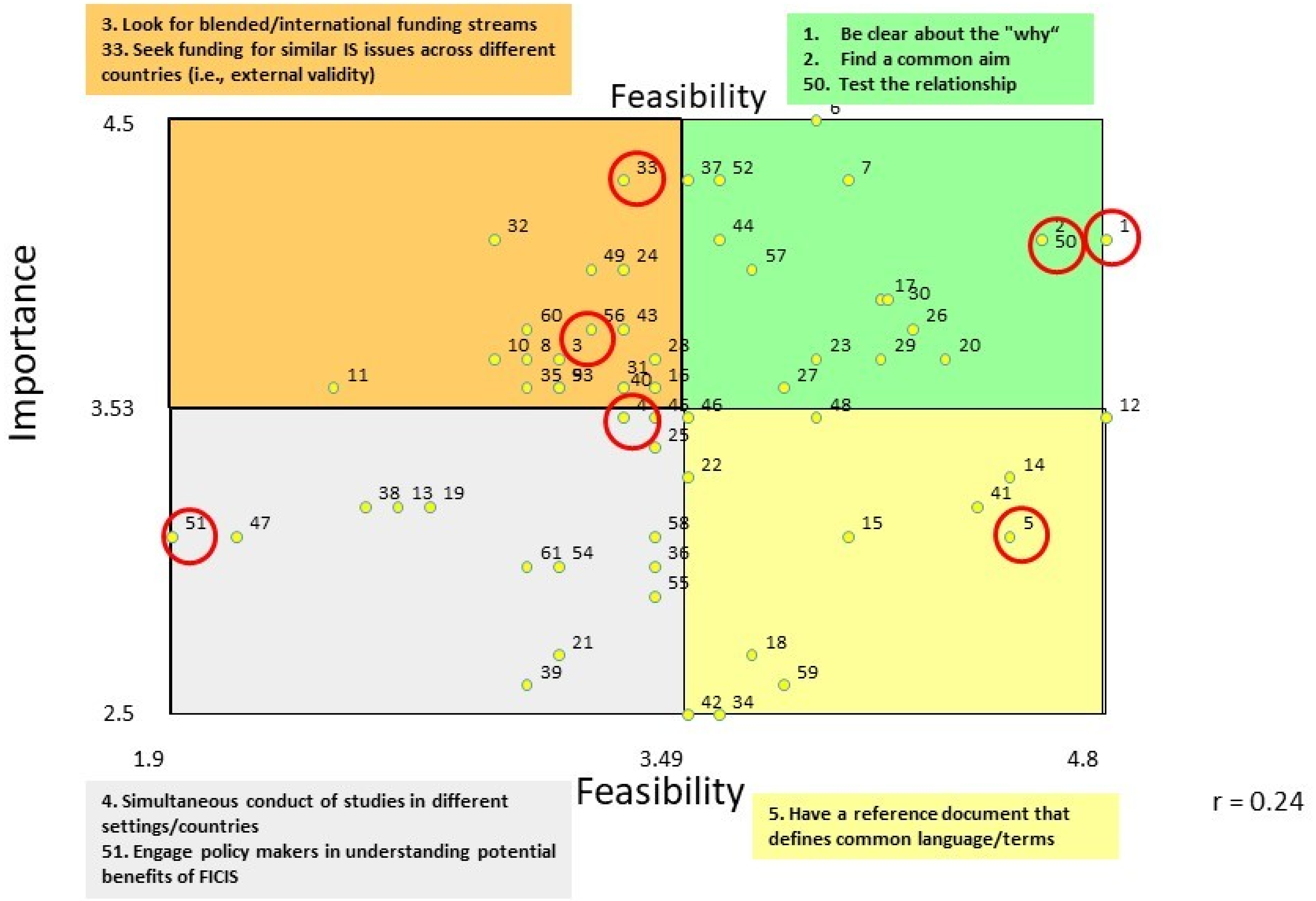
3. Look for blended/international funding streams
33. Seek funding for similar IS issues across different countries (i.e., external validity)
1. Be clear about the "why"
2. Find a common aim
50. Test the relationship
Feasibility
Importance
4.5
3.53
2.5
1.9
3.49
4.8
Feasibility
4. Simultaneous conduct of studies in different settings/countries
51. Engage policy makers in understanding potential benefits of FICIS
5. Have a reference document that defines common language/terms
r = 0.24
