# Supplementary material for: Fostering international collaboration in implementation science and research: a concept mapping exploratory study
Source: BMC Res Notes. 2019 Nov 29;12:778. doi: 10.1186/s13104-019-4800-4 (PMC6883596; doi:10.1186/s13104-019-4800-4)
Supplement: Supplementary file 2 — Additional file 2. List of statements within each cluster. [file 13104_2019_4800_MOESM2_ESM.pdf]

Additional File 2: List of Statements within Each Cluster

Cluster	Statement Number	Statement
1. Strategic Planning	1	Be clear about the "why"
	2	Find a common aim
	17	Developing shared principles for collaboration (e.g., remaining mindful of "positionalities" personalities and reasons for collaboration)
	29	Structured timeline for collaboration, goals, products
	30	Structured plan for collaboration, goals, products
2. Practicality	6	Identify collaborators with common interests
	16	Develop collaboration at early stages in IS idea development (e.g., co-creation) rather than tacking on people
	24	Researchers seeking out international collaborators with expertise needed for specific projects
	28	Identify incentives that will result in benefits relevant to collaborators across countries
	37	Use similar measures/metrics across countries
	45	Mechanisms for announcing interest in having international collaborators
	48	Focus on identifying collaborators who work well together
	50	Test the relationship (e.g., start with something concrete (e.g., write article together and see how the collaboration is working)
3. Define Common Principles	5	Have a reference document that defines common language/terms
	22	Developing template agreement for data sharing/publications across international settings
	25	Open access to measures to promote appropriate translation
	59	Promote principles of diversity and inclusion in international IS
4. Technological Tools for Collaboration	8	Build infrastructure to facilitate easy exchange of resources (e.g., literature, measures, etc.)
	38	Creation of searchable database of IS researchers across countries, interests areas
	41	Use video conferencing to keep international collaborators engaged
	42	Use apps to foster communication among international collaborators (e.g., Slack, What's App, etc.)

5. Funding	3	Look for blended/international funding streams
	7	Be proactive about pursuing funding opportunities for joint knowledge exchange/production
	11	Government sponsored mechanisms for international work (e.g., Fogarty International Center)
	32	Write applications together with co-funding across countries
	33	Seek funding for similar IS issues across different countries (i.e., external validity)
	40	Resource listing key funding that could be used for international IS
6. Disseminate Importance of FICIS	35	Have journals to encourage international perspectives in IS
	36	Special issue on international collaboration in IS
	51	Engage policy makers in understanding potential benefits of international collaborations in IS
7. Knowledge Sharing	39	Resource that identifies key publications in international IS
	53	More open access data and data sharing across international IS studies
	55	Sharing international perspectives on engagement in IS across countries
8. Innovative & Adaptive Research	4	Simultaneous conduct of studies in different settings/countries
	9	Comparative analysis of systems and settings (understanding differences/or not)
	34	Bibliometric study on the level of international empirical research (e.g., country affiliation, settings, funding)
	43	Develop and test new and innovative IS methods (e.g., measures, strategies, etc.) in diverse settings
	49	Enhancing adaptability/generalizability of implementation strategies across countries (core elements and adaptation)
9. Training IS Researchers	10	Establish a rotation program for implementation scientists (PhD, Post-doc, scholars)
	13	Rotating International training program across countries
	21	Identify mechanisms to get newcomers into international IS
	27	Link senior and junior researchers across countries in publications
	44	Involve more senior/expert IS researchers in grants with junior researchers from other countries
	47	Training programs that facilitate Visas for work in other countries
	52	Invite international colleagues as visiting faculty in IS

	54	Training for IS researchers in issues that may be unique in particular countries
	56	Cross country PhD student and post-doc supervision
	57	Engage students, post-docs, in projects in other countries (e.g., using measures/methods of interest)
	60	International guest professorships
	61	Develop ways to get newcomers involved in international IS
10. Networking & Shared Identity	12	Joint symposia presentations with presenters from multiple countries
	14	Small group virtual meetings across countries
	15	Make local lectures/seminar available via web
	26	Promote informal social interaction/meetings of collaborators
	31	Develop consortium for international IS
	58	International networks for specific groups in IS at different career levels (e.g., students, post-docs)
11. Training Support	18	Start an international special interest group (SIG)
	19	Have one or more international conferences
	20	Have parallel meetings with other conferences (e.g., US D&I, Nordic IC, AIC)
	23	Conferences with global focus (e.g., GIC)
	46	Workshop series across countries in international IS
